# Immune-Related miRNA-195-5p Inhibits the Progression of Lung Adenocarcinoma by Targeting Polypyrimidine Tract-Binding Protein 1

**DOI:** 10.3389/fonc.2022.862564

**Published:** 2022-05-05

**Authors:** Lincan Duan, Juan Wang, Dahang Zhang, Yixiao Yuan, Lin Tang, Yongchun Zhou, Xiulin Jiang

**Affiliations:** ^1^ Department of Thoracic Surgery, The Third Affiliated Hospital of Kunming Medical University, Kunming, China; ^2^ Molecular Diagnostic Center, The Third Affiliated Hospital of Kunming Medical University, Kunming, China; ^3^ Key Laboratory of Animal Models and Human Disease Mechanisms of Chinese Academy of Sciences & Yunnan Province, Kunming Institute of Zoology, Kunming, China

**Keywords:** LUAD, miRNA-195-5p, PTBP1, immune cell infiltration, drug sensitivity

## Abstract

**Purpose:**

Lung adenocarcinoma (LUAD) is the most common type of cancer and the leading cause of cancer-related death worldwide, resulting in a huge economic and social burden. MiRNA-195-5p plays crucial roles in the initiation and progression of cancer. However, the significance of the miRNA-195-5p/polypyrimidine tract-binding protein 1 (miRNA-195-5p/PTBP1) axis in the progression of lung adenocarcinoma (LUAD) remains unclear.

**Methods:**

Data were collected from The Cancer Genome Atlas (TCGA) and Gene Expression Omnibus (GEO) databases. The starBase database was employed to examine the expression of miRNA-195-5p, while the Kaplan–Meier plotter, UALCAN, and Gene Expression Profiling Interactive Analysis (GEPIA) databases were utilized to analyze the tumor stage and prognostic value of miRNA and PTBP1. Quantitative reverse transcription-polymerase chain reaction assay was conducted to detect the expression levels of miRNA-195-5p in LUAD cell lines and tissues. The effects of miRNA-195-5p on cell proliferation and migration were examined using the cell growth curve, clone information, transwell assays, and wound healing assays.

**Results:**

We found that miRNA-195-5p was down-regulated in LUAD cancer and cell lines. Importantly, its low levels were related to the tumor stage, lymph node metastasis, and poor prognosis in LUAD. Overexpression of miR-195-5p significantly inhibited cell growth and migration promotes cell apoptosis. Further study revealed that PTBP1 is a target gene of miRNA-195-5p, and overexpression of miRNA-195-5p inhibited the progression of LUAD by inhibiting PTBP1 expression. MiRNA-195-5p expression was related to immune infiltration in lung adenocarcinoma. Moreover, PTBP1 was negatively correlated with diverse immune cell infiltration and drug sensitivity.

**Conclusion:**

Our findings uncover a pivotal mechanism that miRNA-195-5p by modulate PTBP1 expression to inhibit the progression of LUAD. MiRNA-195-5p could be a novel diagnostic and prognostic molecular marker for LUAD.

## Introduction

Lung cancer is one of the most common types of cancer and one of the leading causes of cancer-related death worldwide (1). It mainly includes small cell lung cancer (SCLC) and non-small cell lung cancer (NSCLC); the latter comprises adenocarcinoma (LUAD), squamous cell carcinoma (LUSC), and large-cell carcinoma (LCLC) (2, 3). In recent years, numerous advances in the diagnosis and treatment of LUAD have been achieved. Nevertheless, the incidence and mortality rates of lung cancer remain very high. Therefore, the development of novel therapeutic strategies to improve patient survival time with lung cancer is urgently warranted.

MicroRNAs (miRNAs) are endogenously expressed ncRNAs with important biological function as a posttranscriptional gene regulator (4). Emerging evidence has demonstrated that miRNAs plays crucial roles in cancer initiation, progression, metastasis, and response to therapy (5). MiR-212-3p, miR-27a-3p and miR-132-5p are significantly upregulated in LUAD adenocarcinoma (6). miR-197-5p, miR-93-5p, miR-378a-3p and miR-98-5p downregulate the expression of FUS1/TUSC2, another tumor suppressor gene located on Chr.3p21.3 (7). It has been reported that miR-195-5p is a key regulator in multiple types of cancer [e.g., gastric (8), colorectal (9), cervical (10), prostate (11), and pancreatic (12)]. For instance, it has been confirmed that miR-195-5p modulates the polarization of M2-like tumor-associated macrophages and inhibits the progression of colorectal cancer (9). By regulating the expression of Yes1-associated transcriptional regulator (YAP1), miR-195-5p inhibits the malignant progression of cervical cancer (10). Moreover, it regulates the multi−drug resistance of gastric cancer cells by modulating the expression of zinc finger protein 139 (ZNF139) (13). A previous study showed that miR-195-5p may act as a prognostic factor of the diagnosis of lung cancer (14). However, the potential roles of the miRNA-195-5p/polypyrimidine tract-binding protein 1 (miR-195-5p/PTBP1) axis in modulating LUAD progression remain unclear.

PTBP1, as a significant RNA-binding protein, plays indispensable roles in RNA metabolism (15). It is well documented that abnormal PTBP1 expression leads to progression in various types of cancer. For example, it has been shown that PTBP1 modulates pyruvate kinase M1/M2 (PKM1/M2) splicing, thereby inhibiting cancer-specific energy metabolism (16). In bladder cancer, splicing factor PTBP1 elevated the expression of oncogenic splice variants, predicting poor prognosis (17). High expression of PTBP1 regulates the alternative splicing of cortactin and promotes the progression of colorectal cancer (18). Furthermore, a study suggested that PTBP1 plays important role in the maintenance of growth and malignant properties of breast cancer cells (18). Another crucial function of PTBP1 is its participation in the internal ribosome entry site-mediated translation (19). Indeed, it has been reported that PTBP1 may play indispensable roles in the initiation of translation (20). However, there are no studies investigating its prognostic and immunological significance in patients with LUAD, and the upstream modulatory molecular mechanism of PTBP1 involved in LUAD progression remains unknown.

The objective of this study was to investigate the clinical significance and immunological role of the miRNA-195-5p/polypyrimidine tract-binding protein 1 (miRNA-195-5p/PTBP1) axis in the progression of LUAD.

## Methods

### Data Collection

TCGA-LUAD cohort data and corresponding clinical information of 535 LUAD patients were downloaded from the TCGA website (https://portal.gdc.cancer.gov/repository). The gene expression profiles were normalized using the scale method provided in the “limma” R package. Data analysis was performed with the R (version 3.6.3) and ggplot2 [3.3.3] packages.

### Correlation Between PTBP1 Expression, Immune Cell Infiltrates, and Drug Sensitivity

The TIMER (https://cistrome.shinyapps.io/timer/) (21) and TISIDB (http://cis.hku.hk/TISIDB/) (22) databases were using to examined the correlation between the PTBP1 expression and diverse immune cell infiltrates. We employed the CTRP databases (http://portals.broadinstitute.org/ctrp/) to analysis the correlation between PTBP1 expression and drug sensitivity (23, 24).

### Predicted and Analysis the Target Gene of miRNA-195-5p

We employed the starbase (http://starbase.sysu.edu.cn/), targetscan (http://www.targetscan.org/)and miRDB (http://mirdb.org/) to Predicted and analysis the target gene of miRNA-195-5p (25, 26). Additionally, we using the starbase to examined the correlation and binding site between the PTBP1, miRNA-195-5p.

### Gene Set Enrichment Analysis

We used starbase (https://starbase.sysu.edu.cn/) (27) to obtain the target genes of miRNA-195-5p.

The GO and Kyoto Encyclopedia of Genes and Genomes (KEGG) pathway enrichment analyses were performed for the target gene of miRNA-195-5p using the clusterProfiler package (28).

### Univariate and Multivariate Cox Regression Analyses

Cox regression analysis, including univariate, and multivariate analyses, was used to examine the prognostic value of miRNA-195-5p and PTBP1 in LUAD. The forest plot was constructed using the R package “forest plot” to exhibit the hazard ratio (HR), 95% CI, and p-value.

### Plasmids Construction and Cell Culture

The BEAS-2B cell line was purchased from cell bank of Kunming Institute of Zoology, and cultured in BEGM media (Lonza, CC-3170). Lung cancer cell lines, including A549, H1650 and H1975 were purchased from Cobioer, China with STR document, A549, H1650 and H1975 cells were all cultured in RPMI1640 medium (Corning) supplemented with 10% fetal bovine serum (FBS) and 1% penicillin/streptomycin.

### Quantitative Real-Time PCR

The qRT-PCR assay was performed as documented (29). The primer sequences are list follows: miR-195-5p-F: GAATTCGCCTCAAGAGAACAAAGTGGAG, miR-195-5p R: AGATCTCCCATGGGGGCTCAGCCCCT; U6-F: GGTCGGGCAGGAAAGAGGGC, U6-R: GCTAATCTTCTCTGTATCGTTCC, PTBP1-F: AGCGCGTGAAGATCCTGTTC, PTBP1-R: CAGGGGTGAGTTGCCGTAG, β-actin-F: CTTCGCGGGCGACGAT, β-actin-R: CCATAGGAATCCTTCTGACC. The expression quantification was obtained with the 2−ΔΔCt method.

### Cell Proliferation and Migration Assay

For cell proliferation assay, indicated cells were plated into 12-well plates at a density of 1.5×104, the cell numbers were subsequently counted each day using an automatic cell analyzer countstar (Shanghai Ruiyu Biotech Co., China, IC 1000). For colony formation assay, indicated cells were seeded in 6-well plate (China, NEST, Cat. 703001) with 600 cells per well supplemented with 2 mL cell culture medium, and the cell culture medium was changed every 3 days for 2~3 weeks, and then indicated cells were fixed with 4% PFA and stained with 0.5% crystal violet.

### Clinical Sample Collection

A total of 15 NSCLC patients tissues and adjacent tissues were collected to perform the qRT-PCR assay to examine the expression of miRNA-195-5p in lung cancer. This study was reviewed and approved by Ethics Committee of The Third Affiliated Hospital of Kunming Medical University. This study complies with the ethics committee regulations and conducted with the informed consent of the patients. Meanwhile, these samples were frozen in liquid nitrogen immediately and stored at −80°C for subsequent experiments. The clinical information in **Supplementary Table 5**.

### Statistical Analysis

Correlation analysis was performed using the Pearson correlation test. The significance of the data between the two experimental groups was determined by Student’s t-test, and multiple group comparisons were analyzed by one-way ANOVA. P < 0.05 (*), P < 0.01 (**) and P < 0.001 (***), were considered significant.

## Results

### Pan-Cancer Analysis Revealed Low miRNA-195-5p Expression

**Figure 1:**
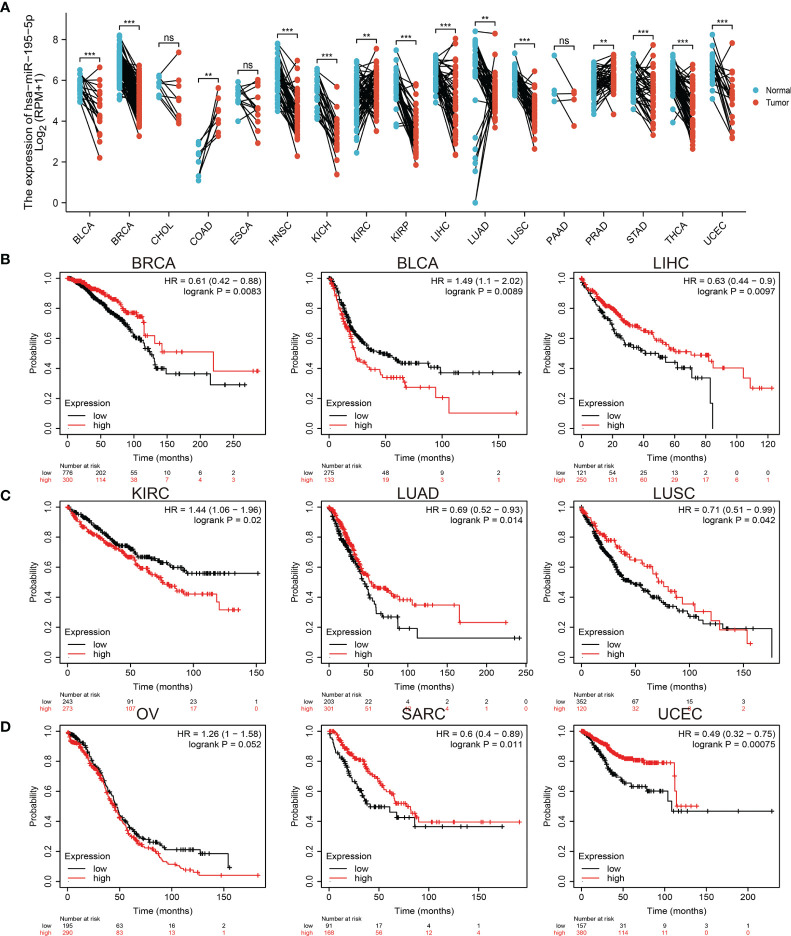
Expression analysis for miRNA-195-5p in human cancers. **(A)** The expression of miRNA-195-5p in pan-cancer analysis by the starbase. **(B–D)** The overall survival of miRNA-195-5p in pan-cancer examine by the kmplot database. **P < 0.01; ***P < 0.001; NS, p > 0.05.

We employed the TCGA database to examine the expression profile of miRNA-195-5p in various human cancers. The results revealed that miRNA-195-5p was low in bladder urothelial carcinoma (BLCA), breast invasive carcinoma (BRCA), head and neck squamous cell carcinoma, kidney chromophobe, kidney renal clear cell carcinoma (KIRC), kidney renal papillary cell carcinoma, liver hepatocellular carcinoma (LIHC), lung adenocarcinoma (LUAD), lung squamous cell carcinoma (LUSC), stomach adenocarcinoma, thyroid carcinoma, and uterine corpus endometrial carcinoma (UCEC) (**Figure 1A** and **Supplementary Table 1**). In contrast, high expression of miRNA-195-5p was observed in KIRC and prostate adenocarcinoma (**Figure 1A**). Collectively, these results demonstrated that the expression of miRNA-195-5p is down-regulated in most human cancers.

Next, to evaluate the prognostic value of miRNA-195-5p, we performed overall survival (OS) analysis in various human cancers. The results showed that low expression of miRNA-195-5p was related to good overall survival (OS) in BLCA, KIRC, and ovarian serous cystadenocarcinoma, and associated with poor overall survival in BRCA, LIHC, LUAD, LUSC, sarcoma, and UCEC (**Figures 1B**–**D**). These results confirmed that miRNA-195-5p may play different roles in the progression of an array of cancers.

### MiRNA-195-5p Expression Was Correlated With the Tumor Stage in Various Cancers

**Figure 2:**
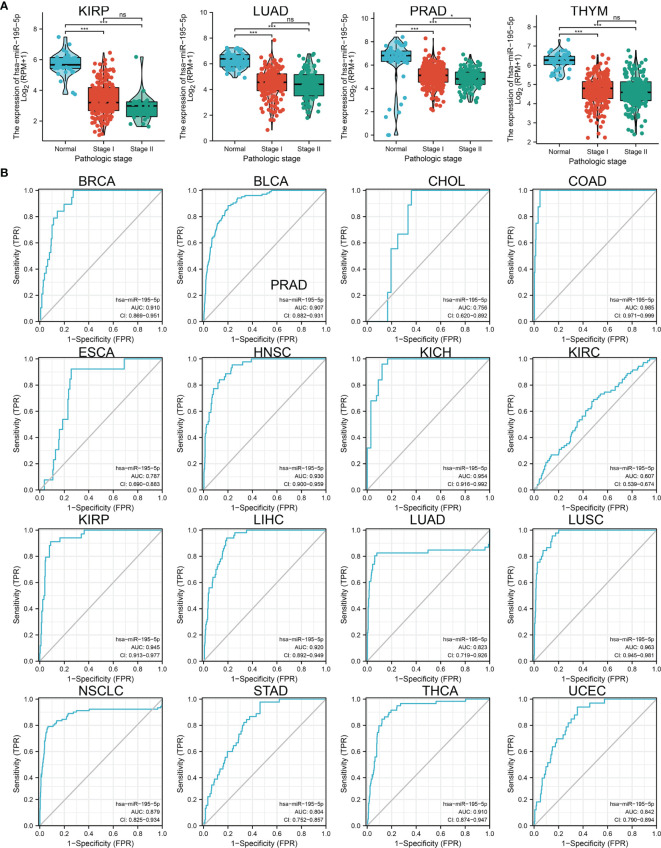
Analysis of the tumor stage and ROC curve for miRNA-195-5p in human cancers. **(A)** The tumor stage for miRNA-195-5p in KIRP, LUAD, PRAD, and THYM. **(B)** ROC curve analyses and AUC values for miRNA-195-5p in diverse human cancer. *P < 0.05; ***P < 0.001; NS, p > 0.05.

Considering its low expression in human cancer, we further explored the correlation between miRNA-195-5p expression and pathological stage in cancer. The results suggested that miRNA-195-5p expression was significantly related to the pathological stage in KIRC, LUAD, PRAD, and THYM (**Figure 2A**). In summary, these findings showed that miRNA-195-5p expression was significantly correlated with the pathological stage of various cancers.

Subsequently, we examined whether miRNA-195-5p acts as a detection index for the diagnosis of various cancers. The receiver operating characteristic curve analysis of miRNA-195-5p showed the following area under the curve (AUC) values: 0.910 for BRCA; 0.907 for BLCA; 0.756 for cholangiocarcinoma; 0.985 for colon adenocarcinoma; 0.787 for esophageal carcinoma; 0.930 for head and neck squamous cell carcinoma; 0.954 for kidney chromophobe; 0.607 for KIRC; 0.945 for kidney renal papillary cell carcinoma; 0.920 for LIHC; 0.823 for LUAD; 0.963 for LUSC; 0.879 for LUAD; 0.804 for stomach adenocarcinoma; 0.910 for thyroid carcinoma; and 0.842 for UCEC (**Figure 2B**). These results indicated that miRNA-195-5p may act as a detection index for the diagnosis of various types of cancer with high sensitivity and specificity.

### Analysis of the Function of Target Genes of miRNA-195-5p

**Figure 3:**
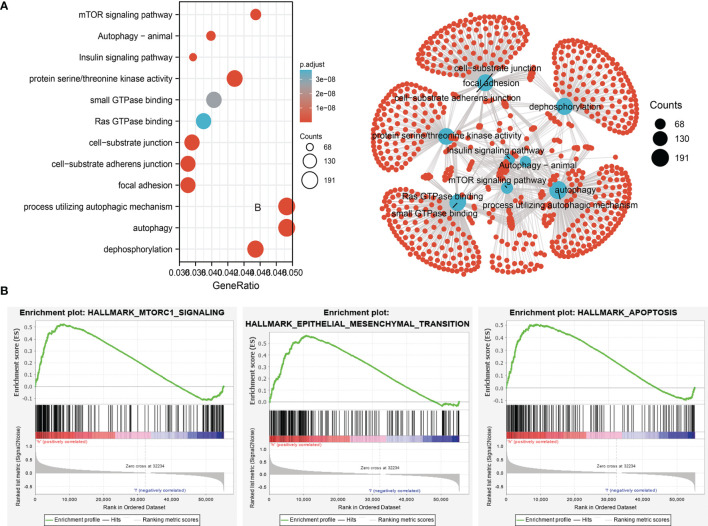
Analysis of the biological function for miRNA-195-5p downstream target genes in human cancers. **(A)**The KEGG signaling pathway of miRNA-195-5p downstream target genes in lung-cancer analysis by the starbase. **(B)** The hallmark of miRNA-195-5p downstream target genes in lung cancer analysis by the GSEA software.

Considering that miRNA-195-5p was markedly correlated with the prognosis, tumor stage, and lymph node metastasis, we next investigated the functions of its target genes in various cancers. We utilized the starBase and TargetScan database to identify the potential target genes of miRNA-195-5p. Next, we used these genes to conducted GO and KEGG enrichment analysis. Results confirmed that these genes were mainly involved in signaling pathways, including the mechanistic target of rapamycin kinase (mTOR), autophagy, and insulin signaling pathways. In terms of molecular function, these target genes are mainly involved in small GTPase binding, Ras GTPase binding, cell-substrate junction, and focal adhesion (**Figure 3A**). The main pathways in which target genes of miRNA-195-5p participate include mTOR signaling, epithelial-mesenchymal transition, and apoptosis (**Figure 3B**). These findings suggested that the target genes of miRNA-195-5p play pivotal roles in cancer progression.

### MiRNA-195-5p Expression Was Down-Regulated in LUAD

**Figure 4:**
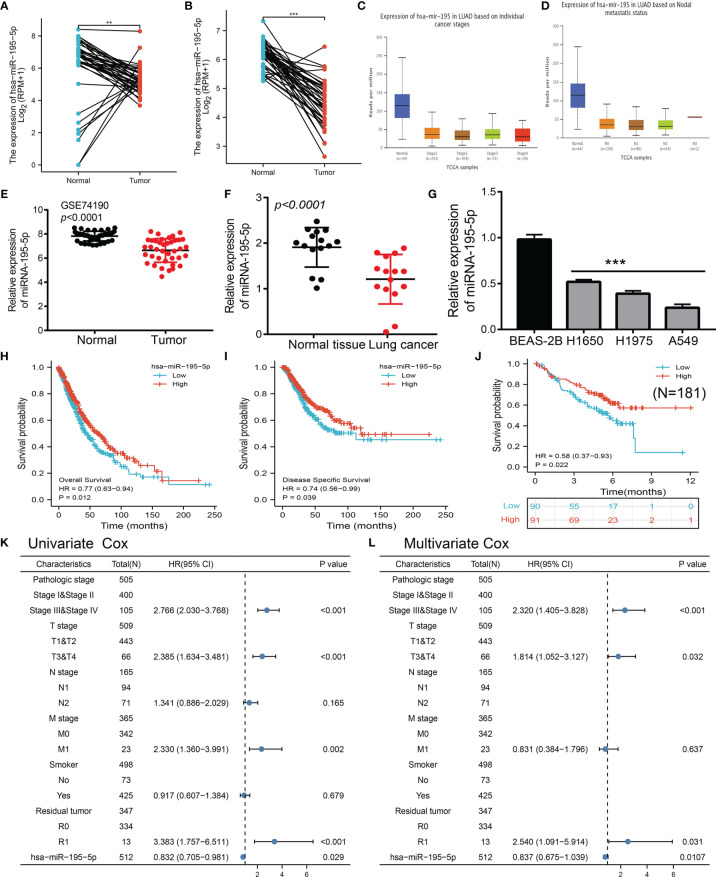
miRNA-195-5p was down-regulated in LUAD and cell lines. **(A, B)**The expression of miRNA-195-5p in lung cancer examine by TCGA datasets. **(C)** The tumor stage of miRNA-195-5p in TCGA-LUAD examine by the UALCAN. **(D)** The lymph node metastases of miRNA-195-5p in TCGA-LUAD examined by the UALCAN. **(E, F)** The expression of miRNA-195-5p in lung cancer determines by GEO datasets. **(G)** The expression of miRNA-195-5p in LUAD cell lines was determined by qRT-PCR assay. **(H–J)**The overall survival and disease-specific survival of miRNA-195-5p in LUAD determine by the TCGA-LUAD dataset and clinical samples. **(K, L)** Univariate and multivariate Cox regression analyses were performed to determine miRNA-195-5p as an independent prognostic factor in the TCGA LUAD dataset. **P < 0.01; ***P < 0.001.

The public database revealed that miRNA-195-5p was significantly decreased in LUAD and LUSC (**Figures 4A, B**); and its low expression correlated with tumor stage and lymph node metastasis (**Figures 4C, D**). To validate the above results, we analyzed a diverse Gene Expression Omnibus (GEO) dataset and found that miRNA-195-5p was decreased in lung cancer tissue (**Figure 4E**). To further investigate the expression of miR-195-5p in LUAD, we first detected its levels in 15 pairs of lung cancer samples using quantitative reverse transcription-polymerase chain reaction (qRT-PCR). The results indicated that miR-195-5p expression was markedly lower in lung cancer tissues compared with adjacent normal tissues (**Figure 4F**). Moreover, we used the qRT-PCR assay to examine the expression of miRNA-195-5p in various LUAD cell lines. The data showed that miRNA-195-5p expression was down-regulated in LUAD cells versus normal BEAS-2B cells (**Figure 4G**). Finally, we found that low expression of miRNA-195-5 was correlated with poor overall survival and disease-specific survival (DSS) in LUAD patients (**Figures 4H, I**); To further validate the overall survival of miR-195-5p in LUAD patients, we examined the prognosis of miR-195-5p in lung cancer by clinical samples from Yunnan Cancer Hospital (N=181). The results also confirmed that low miR-195-5p expression had a worse OS than the high miR-195-5p expression group (**Figure 4J**). These results indicated that miRNA-195-5p may act as a detection index for the diagnosis of LUAD with high sensitivity and specificity.

### MiRNA-195-5p Is an Independent Prognostic Factor in LUAD

To further examine whether miRNA-195-5p was an independent prognostic factor in LUAD. Univariate Cox regression analysis demonstrated that miRNA-195-5p expression (p = 0.029), pathological stage (p < 0.001), T stage (p < 0.001), M stage (p =0.002), and Residual tumor stage (p < 0.001) were significantly correlated with OS in LUAD (**Figure 4K**); Multivariate analysis indicated that miRNA-195-5p expression (p = 0.0107), pathological stage (p < 0.001), T stage (p =0.032), and Residual tumor stage (p=0.031) were significantly correlated with OS in LUAD (**Figure 4L**); These results confirmed that miRNA-195-5p was an independent risk factor for LUAD patients.

### Overexpression of miRNA-195-5p Inhibited the Proliferation and Migration of LUAD Cells

**Figure 5:**
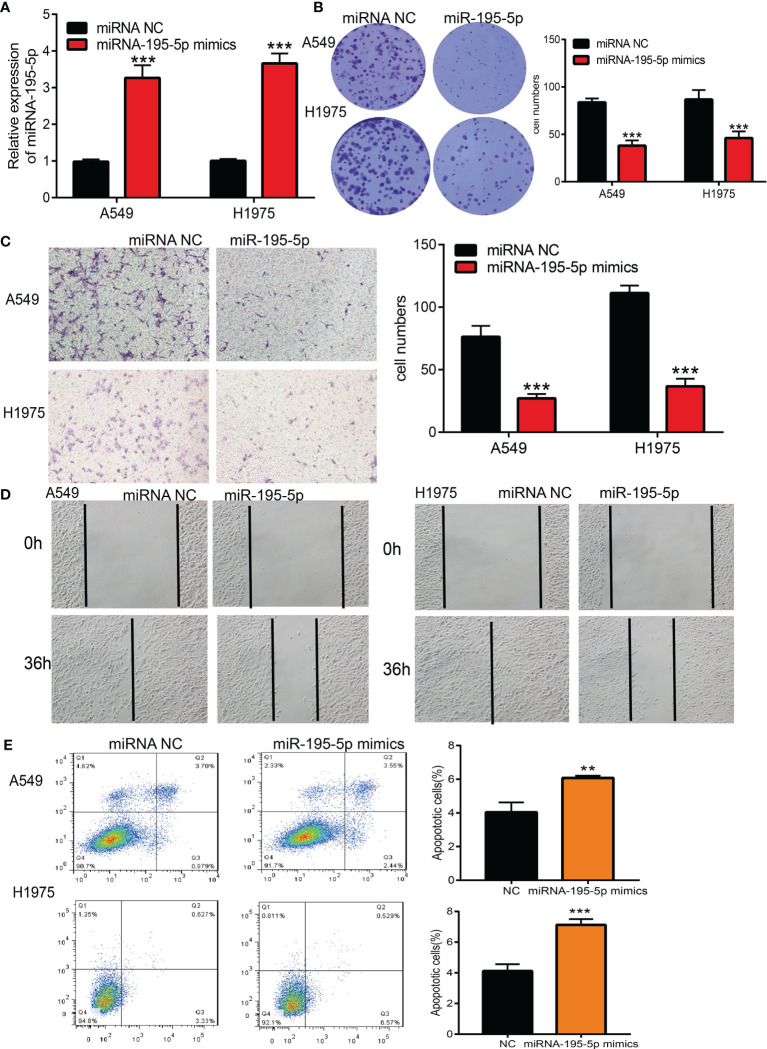
Over-expression of miRNA-195-5p inhibits the cell proliferation and migration ability of LUAD cells. **(A)** The expression of miRNA-195-5p in LUAD cells lines after over-expression of miRNA-195-5p examined by using the qRT-PCR assay. **(B)** Over-expression of miRNA-195-5p on cell growth ability examined by clone information assay. **(C)** Over-expression of miRNA-195-5p on cell migration ability examined by transwell assay. **(D)** Over-expression of miRNA-195-5p on cell migration ability examined by wound healing assay. Quantification data were also indicated. **(E)** Cell apoptosis of LUAD after over expression of miR-195-5p.Scale bar=50 μm. **P < 0.01; ***P < 0.001.

Given the low expression of miRNA-195-5p in LUAD tissues, we speculated that it may play a suppressive role in the pathogenesis of this disease. To investigate its functions in LUAD, we transiently overexpressed miRNA-195-5p mimics in A549 cells. The expression levels of miRNA-195-5p after overexpression were detected using qRT-PCR. As expected, the levels of miRNA-195-5p were increased after overexpression of miR-196b-5p (**Figure 5A**). Furthermore, we conducted the biological function assay to examine the effects of miRNA-195-5p overexpression on the proliferation and migratory ability of NSCLC cells. Results confirmed that overexpression of miRNA-195-5p inhibited the cell growth and cell migration in LUAD, and promoted apoptosis (**Figures 5B**–**E**). Collectively, these data imply that miRNA-195-5p plays tumor-suppressive roles in the progression of lung cancer

### MiRNA-195-5p Exerted Its Effects by Modulating PTBP1 Expression in LUAD

**Figure 6:**
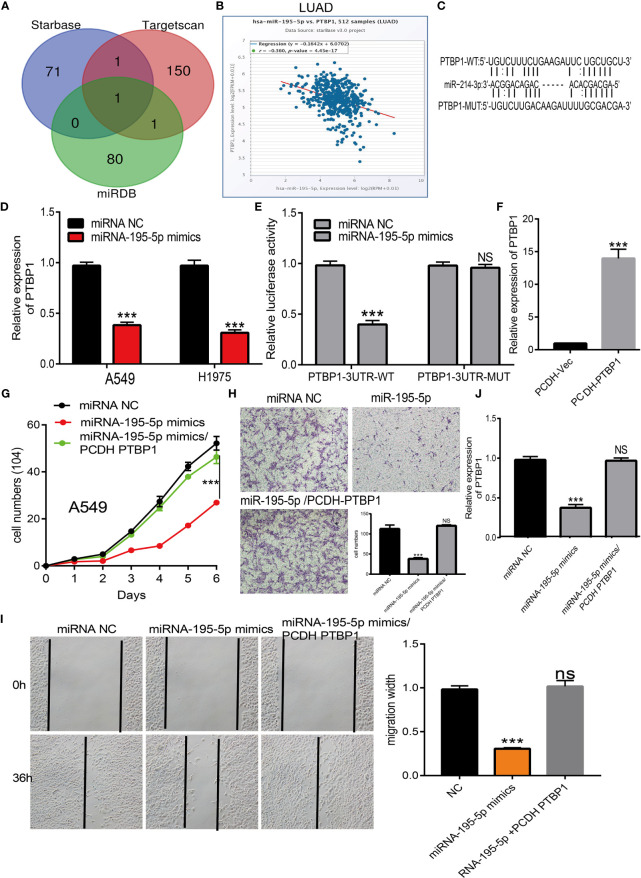
MiR-195-5p modulates the proliferation and migration of LUAD by Targeting PTBP1. **(A)** Identity the PTBP1 as the target gene of miRNA-195-5p by employed diverse public databases. **(B)** The correlation between the miRNA-195-5p and PTBP1 in TCGA LAUD data. **(C)** The target sites between the miRNA-195-5p and PTBP1 were predicted by starBase. **(D)** The expression of PTBP1 in LUAD cell after over-expression of miRNA-195-5p was examined by the qRT-PCR assay. **(E)**The luciferase activities of the PTBP1 luciferase reporter vector (WT or MUT) in A549 cells transfected with miRNA-195-5p mimics or mimics NC. **(F)** The over-expression efficient of PTBP1 in A549 cells examine by qRT-PCR assay. **(G)** Overexpression of PTBP1 could reverse the repressed cell proliferation induced by miR-195-5p overexpression in A549 cells examined by growth curve assay. **(H)** Overexpression of PTBP1 could reverse the repressed cell migration induced by miR-195-5p overexpression in A549 cells examined by transwell assay. **(I)** The expression of PTBP1 in A549 cells after transfection miR-195-5p mimics and over-expression of PTBP1. Quantification data were also indicated. **(J)** Overexpression of PTBP1 could reverse the repressed cell migration induced by miR-195-5p overexpression in A549 cells examined by wound healing assay. Scale bar=50 μm. ***P < 0.001; NS, p > 0.05.

To decipher the underlying mechanism involved in the function of miRNA-195-5p, we performed bioinformatics analyses for the identification of possible targets. We employed the starBase, TargetScan, and miRDB databases to predict downstream genes. The results indicated that PTBP1, as an important lung cancer stem cell marker gene, may be a target gene of miRNA-195-5p (**Figure 6A**). The analysis revealed that miRNA-195-5p was negatively correlated with PTBP1 in lung cancer (**Figure 6B** and **Supplementary Table 2**). We used the starBase database to examine potential binding sites and analyze the correlation between miRNA-195-5p and PTBP1 (**Figure 6C**). In addition, we conducted an overexpression analysis to investigate the effects of miRNA-195-5p on the expression of target genes. Forced miRNA-195-5p expression significantly decreased the mRNA levels of PTBP1 in A549 cells (**Figure 6D**).

The luciferase assay showed that transfection with miR-195-5p mimics significantly reduced the relative luciferase activity of PTBP1-3′-UTR-wild-type-treated lung cancer cells; however, it did not affect that of PTBP1-3′-UTR-mutation-treated lung cancer cells (**Figure 6E**). Based on the above results, we speculated that miR-195-5p might exert its effects by inhibiting PTBP1 expression. We next overexpressed PTBP1 in A549 cell lines and employed qRT-PCR to validate the efficiency (**Figure 6F**). Growth curve results confirmed that the overexpression of PTBP1 could reverse the repressed cell proliferation induced by miR-195-5p overexpression in A549 cells (**Figure 6G**). Transwell and wound healing assays indicated that PTBP1 overexpression abolished the miR-195-5p-caused suppression of migration in A549 cells (**Figures 6H, I**). Overexpression of miR-195-5p reduced the mRNA levels of PTBP1; these levels were restored following overexpression of PTBP1 (**Figure 6J**). Taken together, these findings confirmed that miR-195-5p acted as a tumor suppressor by modulating PTBP1 in LUAD.

### Correlation Analysis Between miRNA-195-5p Expression and Infiltrating Immune Cells

**Figure 7:**
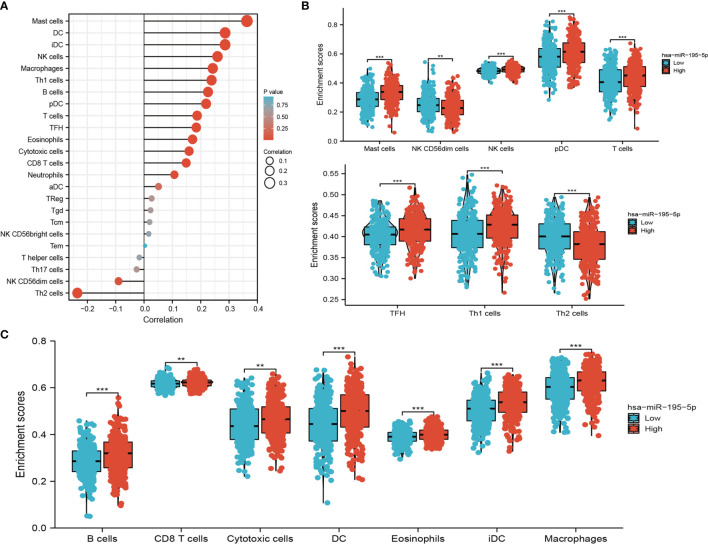
Analysis of the correlation between miRNA-195-5p expression and immune infiltration. **(A)** Correlation between the relative abundances of 24 immune cells and miRNA-195-5p expression level. **(B, C)** Diverse proportions of immune cell subtype in tumor samples in high and low miRNA-195-5p expression groups. **P < 0.01; ***P < 0.001.

Considering miRNA-195-5p plays crucial roles in the progression of lung cancer. We examined the correlation between miRNA-195-5p expression and immune infiltration in LUAD by using Spearman correlation, the analysis data demonstrated that miRNA-195-5p positively correlated with the immune infiltration of Mast cells, DC, iDC, NK cells, Macrophages, Th1 cells, B cells, pDC, T cells, TFH, Eosinophils, Cytotoxic cells, CD8 T cells and Neutrophils, negatively correlated with the immune infiltration of NK CD56dim cells and Th2 cells in LUAD (**Figures 7A**–**C**). These results demonstrated that miRNA-195-5p plays a significant role in the immune response of LUAD.

### PTBP1 Expression Was Upregulated in LUAD

**Figure 8:**
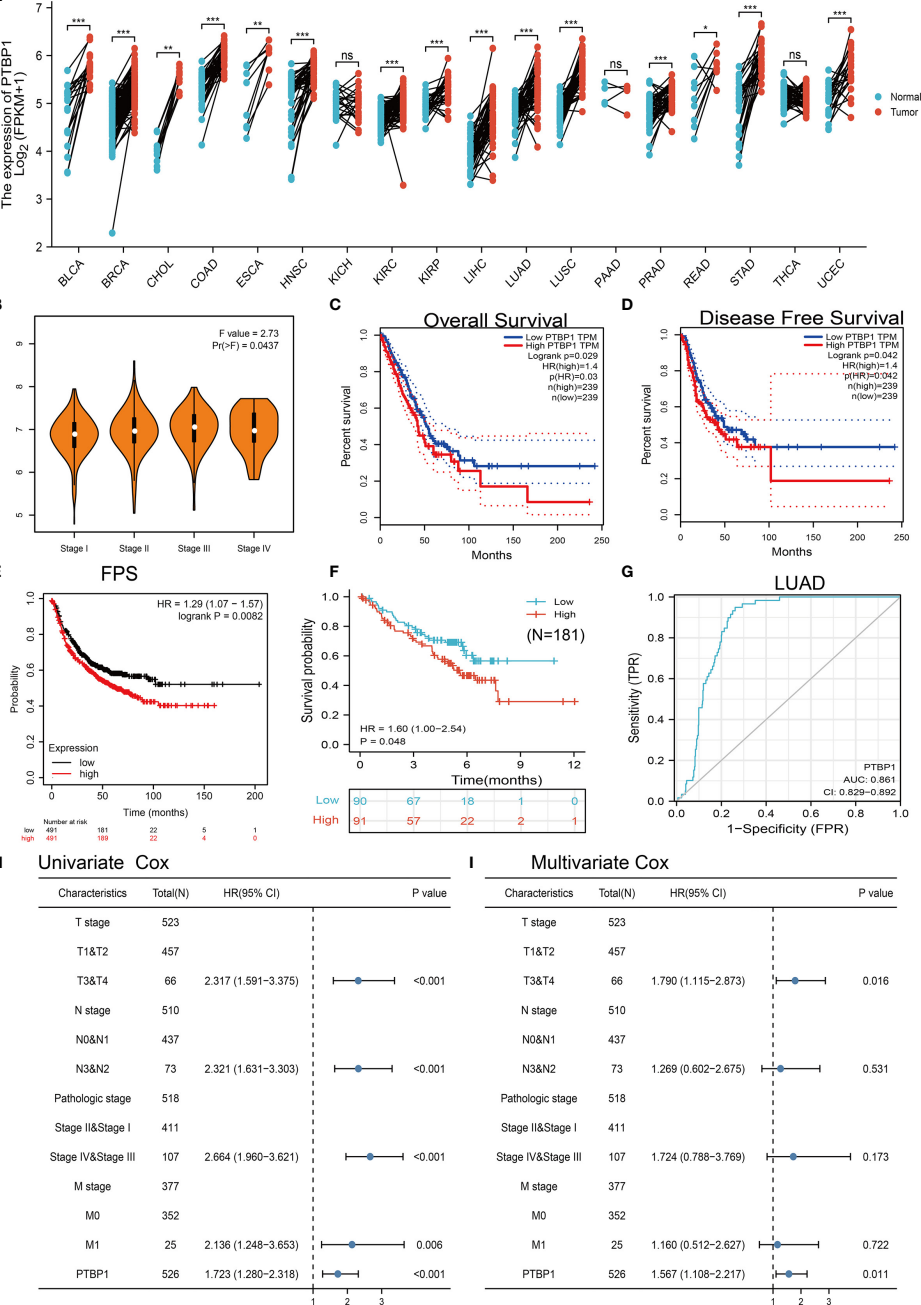
PTBP1 was highly expressed in LUAD. **(A)** The expression of PTBP1 in pan-cancer. **(B)** Analysis of the tumor stage for PTBP1 in TCGA-LUAD analysis by using the GEPIA database. **(C–F)** Analysis of the overall survival, disease-free survival, and progress-free survival for PTBP1 in TCGA-LUAD and clinical sample. **(G)** ROC curve analyses and AUC values for miRNA-195-5p in TCGA-LUAD dataset. **(H, I)** Univariate and multivariate Cox regression analyses were performed to determine PTBP1 as an independent prognostic factor in the TCGA LUAD dataset. *P < 0.05; **P < 0.01; ***P < 0.001; NS, p > 0.05.

We employed the public database and found that PTBP1 was significantly up-regulated in numerous human cancers, particularly LUAD (**Figure 8A**). Moreover, its high expression was correlated with tumor stage and poor prognosis (**Figures 8B–E**). The analysis of GEO datasets yielded consistent results (**Supplementary Table 3**). To further validate the correlation between PTBP1 expression and overall survival, we examined the prognosis of PTBP1 in lung cancer by clinical samples from Yunnan Cancer Hospital (N=181). The results also showed that higher PTBP1 expression had a worse OS than low PTBP1 expression group (**Figure 8F**). The AUC value for LUAD was 0.861 (**Figure 8G**). In addition, higher expression of PTBP1 was correlated with cancer stage (**Supplementary Figure 1**) and poor prognosis (**Supplementary Figure 2**). The receiver operating characteristic curve analysis showed that PTBP1 may act as a detection index for the diagnosis of LUAD with high sensitivity and specificity (**Supplementary Figure 3**). These results demonstrated that PTBP1 plays an important role in tumorigenesis.

### PTBP1 is an Independent Prognostic Factor in LUAD

To examine whether PTBP1 was an independent prognostic factor in cancers, Univariate Cox regression analysis demonstrated that PTBP1expression (p < 0.001), pathological stage (p < 0.001), T stage (p < 0.001), N stage (p < 0.001), and M stage (p = 0.006) were significantly correlated with OS in LUAD (**Figure 8H**); Multivariate analysis indicated that PTBP1 expression (p = 0.011), and T stage (p =0.016) were significantly correlated with OS in LUAD (**Figure 8I**); These results confirmed that PTBP1 was an independent risk factor for LUAD patients lead to adverse clinical outcomes.

### PTBP1 Was Associated With Immune Infiltration in LUAD

**Figure 9:**
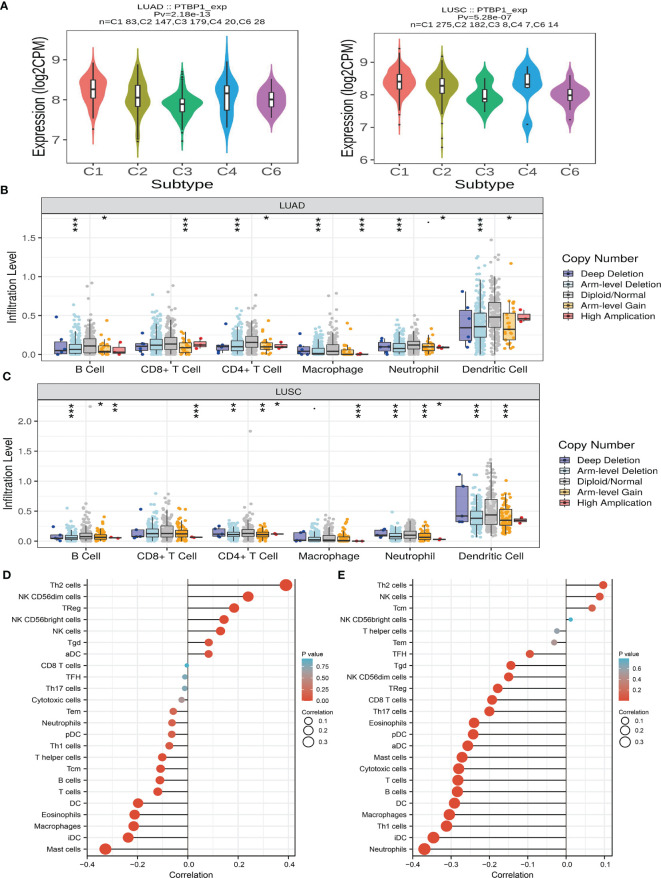
Analysis of the immunological roles of PTBP1 in LUAD. **(A)** The expression of PTBP1 in immune subtype of LUAD. **(B, C)** The correlation between tumor infiltrating levels and somatic copy number alterations of PTBP1 in LUAD. **(D, E)** The association between PTBP1 expression and immune infiltration level in LUAD. *P < 0.05; **P < 0.01; ***P < 0.001.

We analyzed the expression of PTBP1 in immune subtypes of LUAD. The results demonstrated high expression of PTBP1 mainly in the C4 subtype of LUAD and LUSC (**Figure 9A**). Next, analysis of the TIMER database revealed that somatic copy number alterations for PTBP1 were significantly correlated with diverse immune cell infiltration levels in LUAD and LUSC (**Figures 9B, C**). Hence, PTBP1 plays an important role in the immune response, as well as the development and progression of lung cancer. We also explored the correlation between PTBP1 and immune infiltration in LUAD. Results indicated that PTBP1 was positively correlated with the immune infiltration of LUAD tumors by T helper 2 (Th2) cells, natural killer (NK) CD56dim cells, regulatory T NK CD56bright cells, and NK cells. Moreover, it was negatively correlated with immune infiltration by neutrophils, dendritic cells (DC), Th1 cells, Th cells, TcmB cells, T cells, eosinophils, macrophages, interstitial DC, and mast cells (**Figure 9D**). Our results demonstrated that PTBP1 was positively correlated with the immune infiltration of LUSC tumors by Th2 cells, NK cells, and central memory T cells. Also, it was negatively correlated with immune infiltration by CD8 T cells, Th17 cells, eosinophils, plasmacytoid DC, activated DC, mast cells, cytotoxic cells, T cells, B cells, DC, macrophages, Th1 cells, interstitial DC, and neutrophils (**Figure 9E**). Collectively, the data indicated that PTBP1 was significantly correlated with immune infiltration in LUAD.

### Analysis of the Function of PTBP1 in LUAD

**Figure 10:**
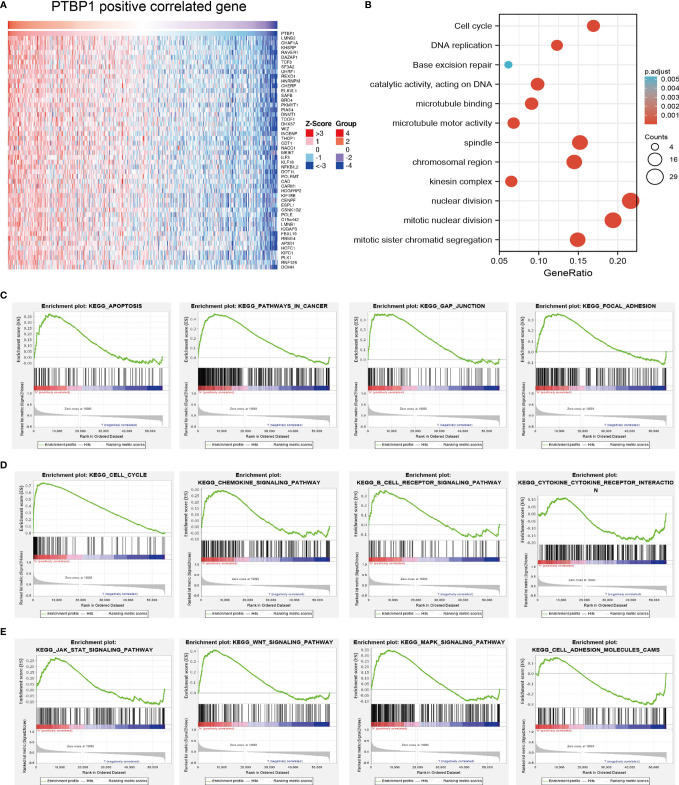
Analysis of the function of PTBP1 in LUAD. **(A)**The positive gene of PTBP1 in lung cancer analysis by employed the Linkedomics tools. **(B)** The KEGG signaling pathway of PTBP1 in lung cancer. **(C–E)** The involvement of genes co-expressed with PTBP1 in LUAD signaling pathways as examined by GSEA software.

We further explored PTBP1-related signaling pathways involved in the progression of LUAD. For this purpose, we employed LinkedOmics to perform a correlation analysis for PTBP1. The heatmap illustrates the genes which were most positively correlated with PTBP1 (**Figure 10A**). We next performed KEGG pathway enrichment. The results showed that up-regulation of PTBP1 expression is mainly involved in cell cycle regulation, DNA replication, and base excision repair (**Figure 10B**). It has been reported that these signaling pathways play important roles in the proliferation of cancer cells.

Gene set enrichment analysis (GSEA) also showed that pathways, including the apoptosis, pathway in cancer, gap junction, focal adhesion, cell cycle, B cell receptor signaling pathway, cytokine-cytokine receptor interaction, chemokine signaling pathway, JAK-STAT signaling pathway, WNT signaling pathway, MAPK signaling pathway, and cell adhesion molecules CAMs, were significantly enriched in the high PTBP1 expression group (**Figures 10C**–**E**).

### Correlation Between PTBP1 Expression and Tumor Mutational Burden, Microsatellite Instability, and Drug Sensitivity

**Figure 11:**
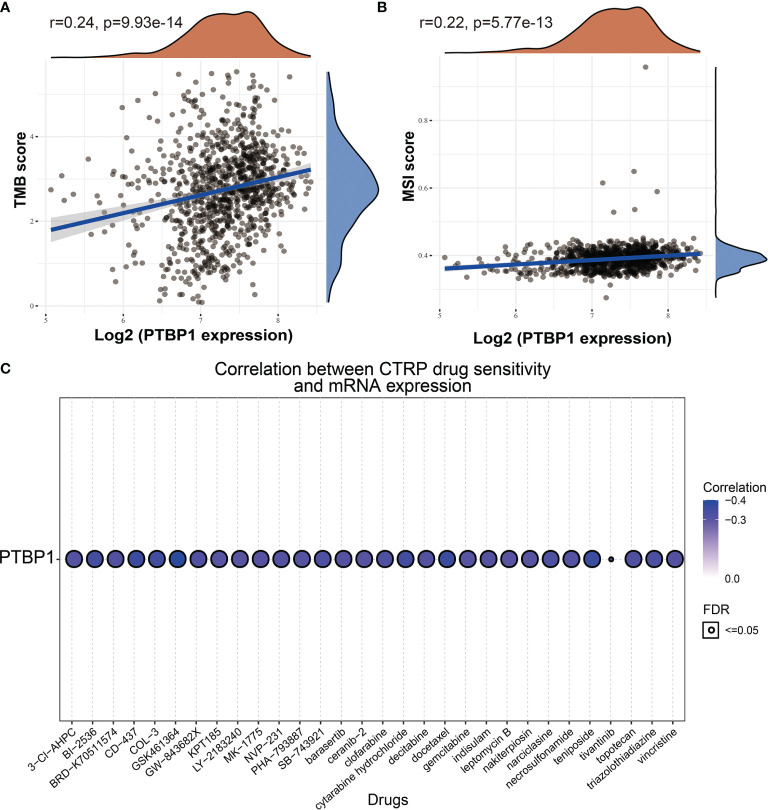
Analysis of the correlation between PTBP1 expression and TMB, MSI and drug sensitivity. **(A)** The correlation between the PTBP1 expression and TMB in LUAD. **(B)** The correlation between the PTBP1expression and MSI in LUAD. **(C)** The correlation between the PTBP1 expression and drug sensitivity.

Emerging evidence has demonstrated that TMB and MSI could be potential biomarkers for predicting the efficacy of immunotherapy for lung cancer (30, 31). The above findings indicated that PTBP1 was significantly correlated with the immune infiltration of tumors. We conducted a correlation analysis to clarify the relationship between PTBP1 expression and TMB, MSI, and drug sensitivity. We found that PTBP1 was also positively correlated with the TMB and MSI in LUAD (**Figures 11A, B**). For the exploration of potential therapeutic targets, it is extremely important to examine the correlation between PTBP1 expression and various drugs in a pan-cancer analysis. In the present study, we employed the Gene Set Context Analysis tools to analyze the relationship between PTBP1 expression and drug sensitivity. The results demonstrated that PTBP1 expression was negatively correlated with sensitivity to GSK-J4, GSK461364, BRD-K30748066, docetaxel, CD-437, teniposide, chemically modified tetracycline-3 (COL-3), cytarabine hydrochloride, BI-2536, tivantinib, triazolothiadiazine, narciclasine, SB-743921, clofarabine GW-843682X, topotecan, BRD-K70511574, bafilomycin A1, vincristine, decitabine, NVP-231, barasertib, necrosulfonamide, indisulam, PHA-793887, MK-1775, and ceranib-2 (r<−0.30) (**Figure 11C** and **Supplementary Table 4**). Taken together, these results suggested that PTBP1 was significantly associated with the sensitivity of different cancer cell lines to various drugs

## Discussion

Despite its high incidence and mortality, the exact cause of the development of LUAD is not fully understood. It is well established that LUAD has diverse pathological features. Accumulating evidence suggests that miRNAs play indispensable roles in cancer progression and drug resistance. However, the molecular mechanisms involved in these processes remain unclear. In this study, we analyzed miR-195-5p expression, prognostic value, target genes, and correlation with tumor immune cell infiltration in LUAD for the first time.

The present findings revealed that miRNA-195-5p expression was significantly decreased in various types of cancer, and its low expression was associated with the tumor stage, lymph node metastasis, and unfavorable prognosis of lung cancer. Indeed, it has been confirmed that miR-195-5p was down-regulated in melanoma (32), oral squamous cell carcinoma (33), colon cancer (34), and hepatocellular carcinoma (35). These studies indicated that miR-195-5p plays a fundamental role in malignant suppression, and our results are consistent with these findings.

The high expression of miR-195 can inhibit tumorigenesis and progression and affect the sensitivity of chemotherapeutic agents for malignant tumors, a property that has been applied to drug development. Therefore, it was considered that propofol might induce miR-195 to inhibit the proliferation, migration, and invasion of gastric cancer cells (36). Zuo et al. investigated miR-195 together with long-chain non-coding 00485 (LINC00485) in cisplatin resistance and found that overexpression of miR-195 or silencing of LINC00485 enhances the sensitivity of lung adenocarcinoma cells to cisplatin (37). In this study, we found that miR-195-5p expression was downregulation in LUAD, compared to normal tissues. Moreover, the miR-195-5p expression has associations with tumor pathological stage, lymph node metastasis in LUAD. Meanwhile, the lower miR-195-5p level was related to lymph node metastasis, high tuimor stage. These results suggest that miR-195-5p plays an important role in the progression decreased in some tumor tissues and is associated with clinicopathological features, including late T stage, lymph nodal metastasis, and TNM staging (38, 39). We also found that miRNA-195-5p by inhibited PTBP1 expression lead to inhibition of the prognosis of LUAD.

Results from survival analysis showed that low miR-195-5p expression was associated with poor os, and DSS in LUAD, consistent with previous findings miR-195-5p affects tumor growth and invasion and leads to a poor prognosis (34). Of note, miRNA-195-5p was found to regulate the expression of oncogenes or tumor suppressors and participated in the development of various types of cancer. By targeting mitofusin 2 (MFN2) and F-box and WD repeat domain-containing 7 (FBXW7), miR-195-5p promotes cardiomyocyte hypertrophy (39, 40). It has been reported that miR-195-5p modulates the expression of forkhead box K1 (FOXK1), thereby inhibiting the proliferation of lung cancer cells (41). Notably, circAGFG1 inhibits the expression of miR-195-5p and promotes the progression of triple-negative breast cancer (42). Although some of these findings lack additional experimental verification, our data indicate that miRNA-195-5p may be a promising target and diagnostic or therapeutic biomarker for LUAD. Collectively, these results demonstrated that miRNA-195-5p can be used as a prognostic biomarker for LUAD.

Cancer progression is a complicated process accompanied by increased proliferation, resistance to cell death, enhanced angiogenesis, escape from immune surveillance, and tumor microenvironment (TME) (43). The TME has attracted wide attention in cancer immunotherapy and has been identified as the main contributor to cancer initiation and development (44). Although immunotherapy has made breakthroughs in cancer treatment, it still faces many challenges, and only a limited proportion of cancer patients respond well to immunotherapy. Therefore, the identification of new targets and biomarkers is the key to further improving the efficacy of immunotherapy. Tumor-infiltrating immune cells, including B cells, T cells, dendritic cells, macrophages, and neutrophils, are the major part of the TME (45). Here, to further estimate the relationships between miRNA-195-5p and the TME, we first examined the correlation of miRNA-195-5p expression and the abundance of different infiltrating immune cells across different cancer types. We found that miRNA-195-5p positively correlated with the immune infiltration of Mast cells, DC, iDC, NK cells, Macrophages, Th1 cells, B cells, pDC, T cells, TFH, Eosinophils, Cytotoxic cells, CD8 T cells, and Neutrophils, negatively correlated with the immune infiltration of NK CD56dim cells and Th2 cells in LUAD. These results demonstrated that miRNA-195-5p plays a significant role in the immune response of LUAD.

PTBP1 or polypyrimidine tract binding protein 1 (hnRNPI), is one of the most investigated RBP in vertebrates involved in almost all steps of mRNA regulation during tumorigenesis, due to its RNA-binding activity. PTBP1 is generally described as a widely expressed factor in adult tissues, and accordingly, it is present in most of the cell lines studied (46). The expression levels of PTBP1 have been found to be elevated in brain tumors (47), and different malignant cell lines (48). Furthermore, high expression of PTBP1 has been demonstrated to be associated with the aggressive behavior of several types of cancer, especially in glioma and ovarian tumors (49).

As an important RNA-binding protein, PTBP1 plays a crucial role in RNA metabolism and the progression of numerous types of cancer. Recent studies suggested that CD154 has anti-tumor activity and growth-inhibitory effects and that PTBP1 plays a crucial role in stabilizing CD154 mRNA (50). PTBP1 can reduce the expression of hypoxia-inducible factor 1α (HIF-1α) by modulating its mRNA stability, thereby inhibiting cell invasion when localized in the cytoplasm (51).

Recently, a study showed that PTBP1 induces the mRNA expression of p19 and promotes the proliferation of LUAD cells (52). Studies focusing on the function of PTBP1 in the modulation of RNA splicing in the nucleus was also found that it may play crucial oncogenic roles in the progression of various cancers. Thus, PTBP1 plays an important role and has different functions in tumorigenesis by regulating the amounts of target genes associated with malignancy.

Our data revealed that PTBP1 was significantly higher in LUAD cancer tissues compared with normal human lung tissue, consistent with previous studies (53). A recent study found that PTBP1 is overexpressed in ovarian tumors and colorectal cancer (54), indicating that PTBP1 is closely associated with the pathogenesis and development of cancer. However, the correlation of PTBP1 with clinical characteristics has not been clarified. Further analysis was performed to investigate the relationship between PTBP1 expression in LUAD tissues and clinicopathological characteristics of cancer. The results revealed that the level of PTBP1 expression in LUAD tissue was positively correlated with pathological stage and adverse clinical outcomes.

Regarding PTBP1, Morrel et al. suggested that PTBP1 might be a target of miR-214, which could reduce the endothelial cell glycolysis by inhibiting PTBP1 (55). Akao et al. found that PTBP1 is a target gene of microRNA-133b in colorectal tumors and that microRNA-133b might reduce the proliferation and invasion ability of COAD by inhibiting PTBP1 (56). In the present study, the PTBP1 expression significantly increased in the LUAD cells with overexpression of miR-195-5p upon and qPCR, while the PTBP1 expression significantly reduced in the A549 cells with inhibited expression of miR-195-5p. *Via* the dual-luciferase reporter assay, PTBP1 was confirmed to be the direct target gene of miR-195-5p, which could improve the cell proliferation and migration of LUAD cells. Additionally, our results suggested that miR-195-5p could have inhibited the cell proliferation and migration of LUAD cells and induced cell apoptosis. Moreover, it was also confirmed that the overexpression of PTBP1 might have promoted cell proliferation and migration in the A549 cells, while miR-195-5p might have inhibited cell proliferation and migration by reducing the PTBP1expression. We also found that PTBP1 expression was significantly correlated with the TMB and MSI in LUAD. PTBP1 expression was also positively or negatively correlated with diverse immune cell infiltration. Finally, our results showed that PTBP1 expression was negatively correlated with sensitivity to numerous drugs. The above findings offer promise for the diagnosis and treatment of lung cancer in the future.

This study improves our understanding of the correlation between miRNA-195-5p and LUAD, but some limitations still exist. First, although we explored the correlation between miRNA-195-5p and immune infiltration in LUAD patients, there is a lack of experiments to validate the function of miRNA-195-5p in the tumor microenvironment regulation of LUAD. Second, we uncover that depletion of miRNA-195-5p was inhibited cell proliferation and cell migration of LUAD cells.

However, the molecular mechanisms of miRNA-195-5p in tumor growth and metastasis need to be explored in further studies. Third, we did not conduct the *in vivo* experiments to validate the function of miRNA-195-5p in the tumor metastasis and tumor microenvironment regulation of LUAD. In the future, we will further study the function of miRNA-195-5p in tumor metastasis and tumor microenvironment regulation of LUAD.

## Conclusion

In summary, our findings demonstrate that downregulation of miRNA-195-5p was associated with poor survival in patients with LUAD, and miRNA-195-5p inhibits growth and invasion of LUAD cells by regulating PTBP1 may provide a critical diagnostic and prognostic molecular marker for LUAD.

## Data Availability Statement

The original contributions presented in the study are included in the article/**Supplementary Material**. Further inquiries can be directed to the corresponding authors.

## Ethics Statement

This study was reviewed and approved by the Ethics Committee of The Third Affiliated Hospital of Kunming Medical University.

## Author Contributions

LD, JW, and DZ designed this work, and performed experiments, YY and LT analyzed data. YZ and XJ wrote and revised the manuscript. All authors have read and approved the final version of the manuscript.

## Funding

This work was supported by the National Nature Science Foundation of China (82160508), Yunnan Applied Basic Research Projects (YNWRMY-2019-067) Yunnan Province Specialized Training Grant for High-Level Healthcare Professionals (D-201614), and Yunnan Province Applied Basic Research Foundation (2019FE001) to LD.

## Conflict of Interest

The authors declare that the research was conducted in the absence of any commercial or financial relationships that could be construed as a potential conflict of interest.

## Publisher’s Note

All claims expressed in this article are solely those of the authors and do not necessarily represent those of their affiliated organizations, or those of the publisher, the editors and the reviewers. Any product that may be evaluated in this article, or claim that may be made by its manufacturer, is not guaranteed or endorsed by the publisher.
