# Supplementary material for: Immune-Related miRNA-195-5p Inhibits the Progression of Lung Adenocarcinoma by Targeting Polypyrimidine Tract-Binding Protein 1
Source: Front Oncol. 2022 May 5;12:862564. doi: 10.3389/fonc.2022.862564 (PMC9117652; doi:10.3389/fonc.2022.862564)
Supplement: Supplementary file 1 [file DataSheet_1.docx]

Supplementary Material

## Supplementary Figures


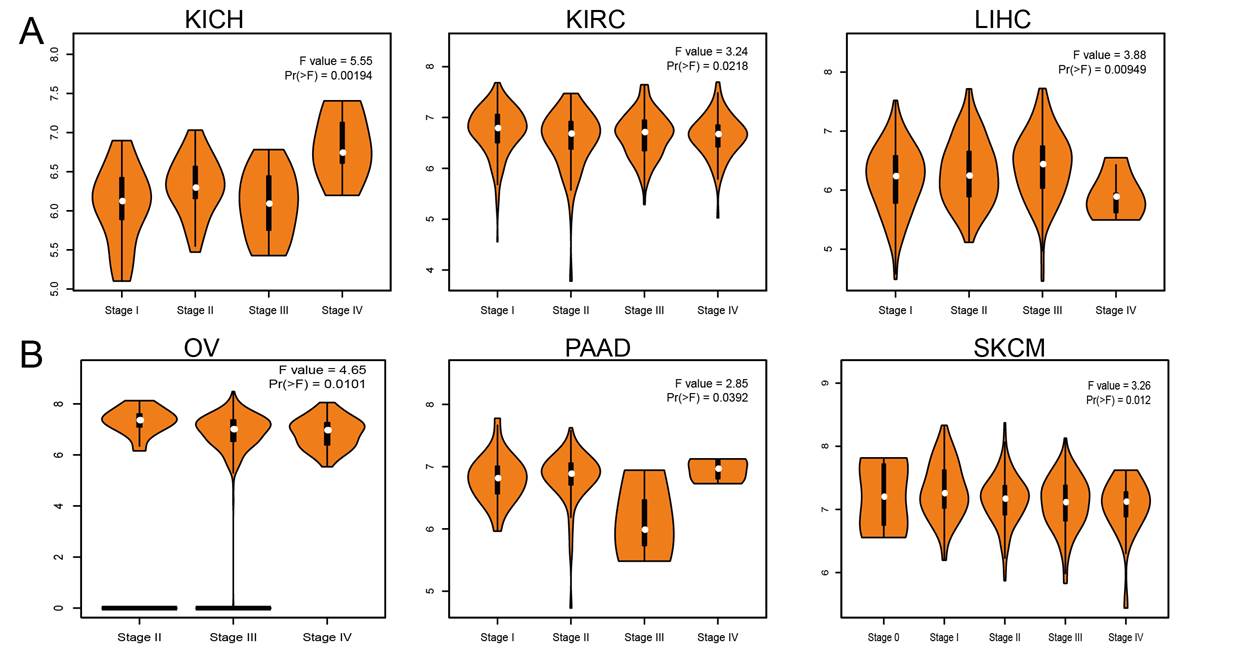


**Supplementary Figure 1.** The tumor stage of PTBP1 in human-cancer. (A) The tumor stage of PTBP1 in KICH, KIRC and LIHC examined by GEPIA database. (B) The tumor stage of PTBP1 in OV, PAAD and SKCM examined by GEPIA database.


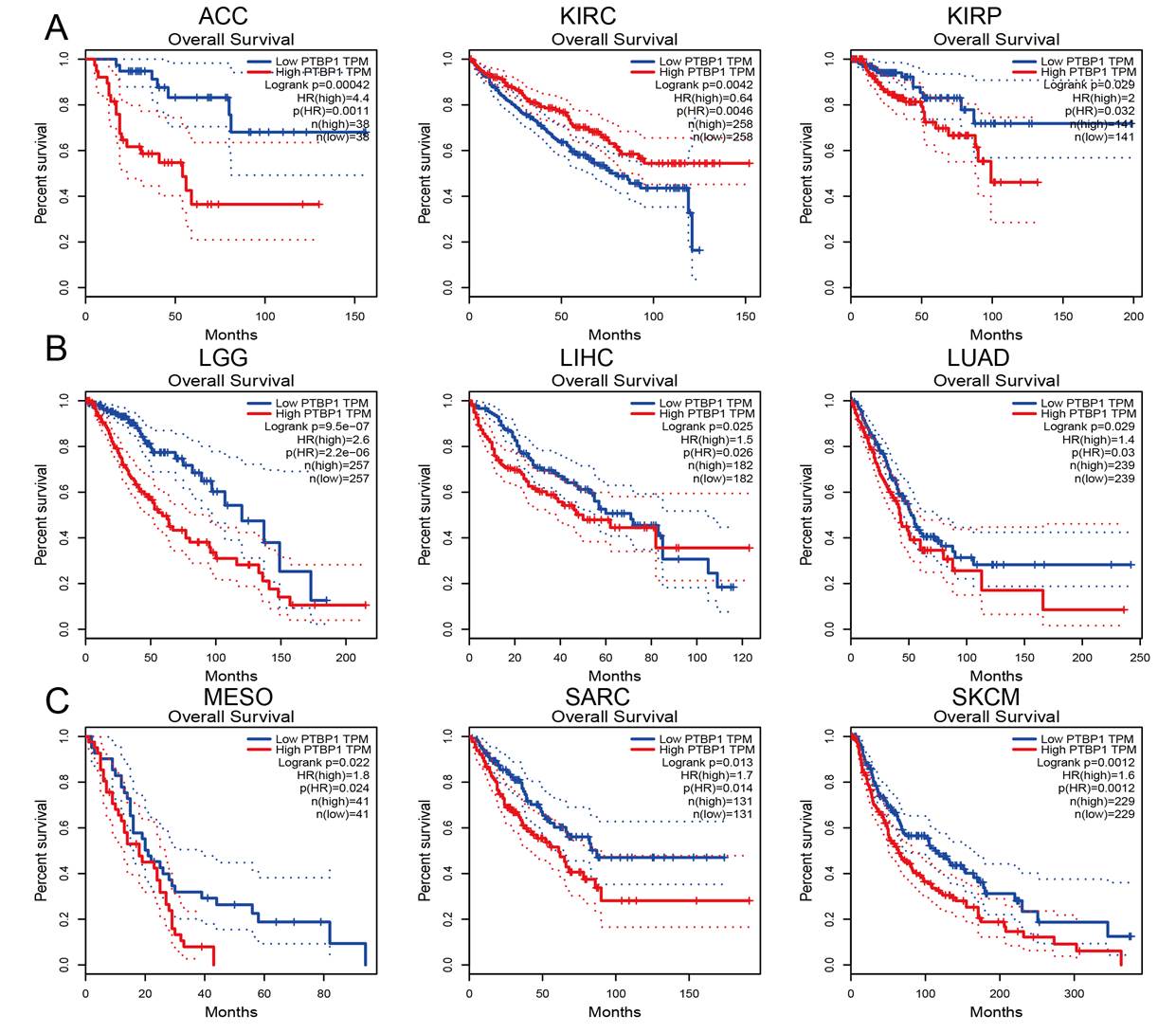


**Supplementary Figure 2.** The prognosis of PTBP1 in human cancer. (A) The prognosis of PTBP1 in ACC, KIRC and KIRP examined by GEPIA database. (B) The prognosis of PTBP1 in LGG, LIHC and LUAD examined by GEPIA database. (C) The prognosis of PTBP1 in MESO, SARC and SKCM examined by GEPIA database.

**
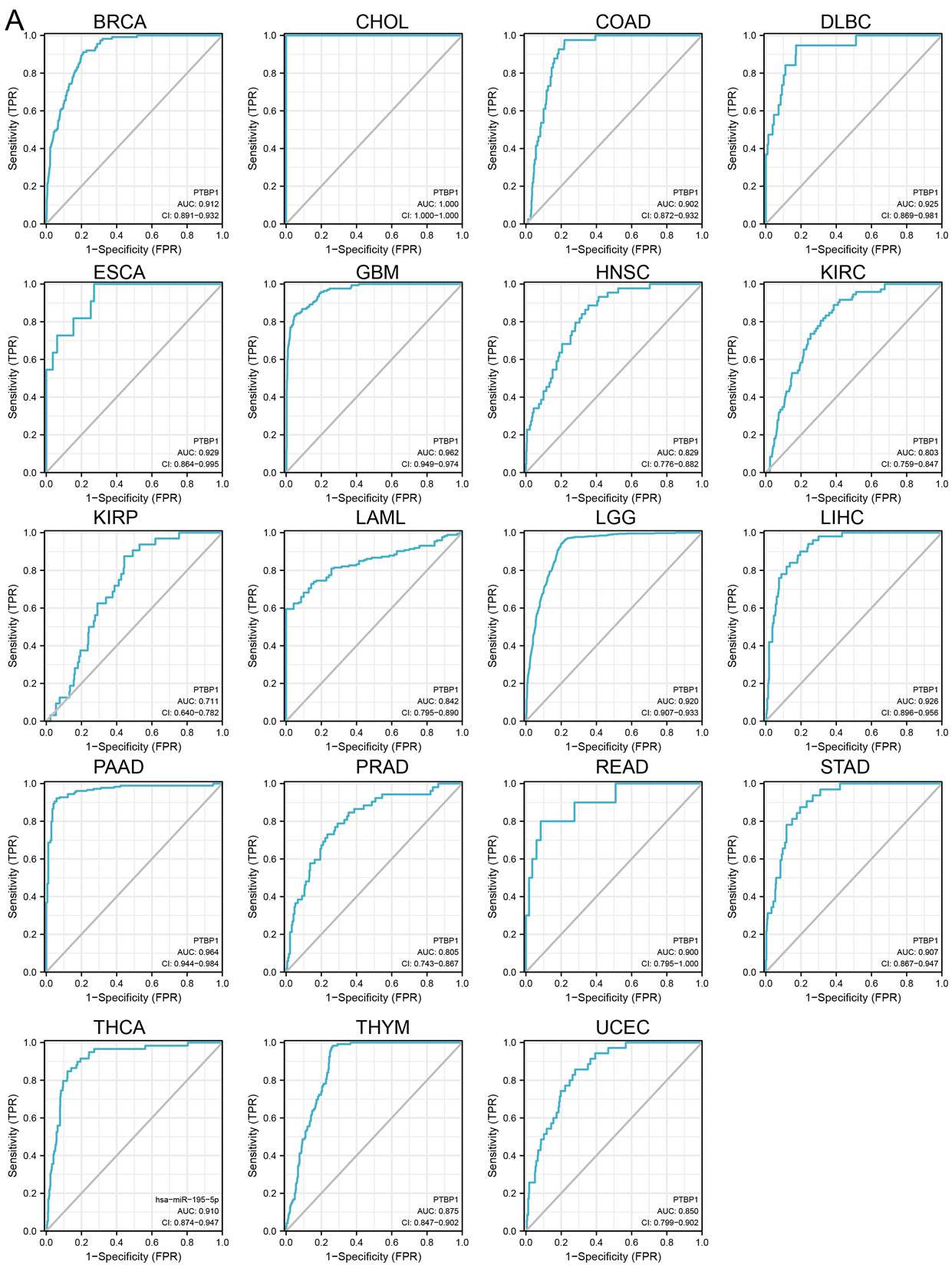
Supplementary Figure 3.** The ROC curve of PTBP1 in diverse human cancer

**Supplementary Table 1. Analysis the expression of miRNA-195-5p in diverse cancer by employed the starbase.**

| CancerFullName | CancerNum | NormalNum | CancerExp | NormalExp | FoldChange | pValue |
| --- | --- | --- | --- | --- | --- | --- |
| BRCA | 1085 | 104 | 44.72 | 114.14 | 0.39 | 2.30E-50 |
| HNSC | 497 | 44 | 22.71 | 76.38 | 0.3 | 1.10E-29 |
| LUSC | 475 | 38 | 27.63 | 76.56 | 0.36 | 3.10E-29 |
| THCA | 509 | 58 | 30.97 | 74.08 | 0.42 | 1.80E-27 |
| KIRP | 289 | 32 | 14.02 | 59.25 | 0.24 | 3.00E-23 |
| LIHC | 370 | 50 | 29.48 | 82.17 | 0.36 | 1.40E-22 |
| KICH | 65 | 24 | 12.32 | 44.78 | 0.28 | 1.50E-14 |
| COAD | 450 | 8 | 27.6 | 3.61 | 7.65 | 2.00E-13 |
| UCEC | 538 | 33 | 55.09 | 136.55 | 0.4 | 9.00E-11 |
| STAD | 372 | 32 | 35.29 | 69.01 | 0.51 | 9.70E-10 |
| BLCA | 408 | 19 | 23.88 | 54.84 | 0.44 | 3.80E-09 |
| KIRC | 517 | 71 | 55.49 | 41.68 | 1.33 | 0.0027 |
| ESCA | 162 | 11 | 25.14 | 37.35 | 0.67 | 0.0072 |
| CHOL | 36 | 9 | 39.48 | 53.08 | 0.74 | 0.035 |

**Supplementary Table 2.The correlation between the miRNA-195-5p expression and PTBP1 in diverse cancer analysis by the starbase database.**

| Cancer | SampleNum | Coefficient-R | p-value |
| --- | --- | --- | --- |
| DLBC | 47 | -0.403 | 4.95E-03 |
| LUAD | 512 | -0.36 | 4.45E-17 |
| STAD | 372 | -0.31 | 1.03E-09 |
| ESCA | 162 | -0.28 | 3.15E-04 |
| MESO | 86 | -0.224 | 3.83E-02 |
| READ | 161 | -0.22 | 5.08E-03 |
| SARC | 261 | -0.195 | 1.55E-03 |
| BLCA | 408 | -0.188 | 1.36E-04 |
| SKCM | 449 | -0.187 | 6.84E-05 |
| LUSC | 475 | -0.11 | 1.60E-02 |
| THCA | 509 | -0.09 | 4.30E-02 |

**Supplementary Table 3. The prognosis of PTBP1 in diverse cancer analysis by the prognoscan database.**

| Dataset | Cancer type | Prognosis | P value |
| --- | --- | --- | --- |
| GSE31210 | Lung cancer | RFS | 1.26E-05 |
| GSE31210 | Lung cancer | RFS | 0.0001 |
| GSE30929 | Soft tissue cancer | DFS | 0.000186 |
| GSE30929 | Soft tissue cancer | DFS | 0.000463 |
| GSE4922 | Breast cancer | DFS | 0.000481 |
| GSE30929 | Soft tissue cancer | DFS | 0.000582 |
| GSE4271 | Brain cancer | OS | 0.000604 |
| GSE2658 | Blood cancer | DSS | 0.00072 |
| GSE30929 | Soft tissue cancer | DFS | 0.001089 |
| GSE30929 | Soft tissue cancer | DFS | 0.001362 |
| GSE31210 | Lung cancer | RFS | 0.002043 |
| GSE13213 | Lung cancer | OS | 0.00284 |
| GSE9891 | Ovarian cancer | OS | 0.002943 |
| GSE7378 | Breast cancer | DFS | 0.003144 |
| GSE4271 | Brain cancer | OS | 0.003199 |
| GSE1456 | Breast cancer | RFS | 0.003842 |
| GSE7378 | Breast cancer | DFS | 0.003893 |
| GSE4271 | Brain cancer | OS | 0.00407 |
| GSE4271 | Brain cancer | OS | 0.005597 |
| GSE1456 | Breast cancer | RFS | 0.005869 |
| GSE16581 | Brain cancer | OS | 0.005955 |
| GSE9893 | Breast cancer | OS | 0.005994 |
| GSE1456 | Breast cancer | RFS | 0.006665 |
| GSE4922 | Breast cancer | DFS | 0.006734 |
| GSE12276 | Breast cancer | RFS | 0.007262 |
| GSE7378 | Breast cancer | DFS | 0.007317 |
| GSE31210 | Lung cancer | RFS | 0.007478 |
| GSE31210 | Lung cancer | OS | 0.00766 |
| GSE13507 | Bladder cancer | DSS | 0.008043 |
| GSE7378 | Breast cancer | DFS | 0.008527 |
| GSE7378 | Breast cancer | DFS | 0.009135 |
| GSE1456 | Breast cancer | DSS | 0.009382 |
| GSE14764 | Ovarian cancer | OS | 0.009775 |
| GSE1456 | Breast cancer | DSS | 0.010066 |
| GSE1456 | Breast cancer | RFS | 0.010083 |
| GSE3494 | Breast cancer | DSS | 0.010517 |
| GSE1456 | Breast cancer | DSS | 0.01119 |
| GSE31210 | Lung cancer | RFS | 0.012373 |
| GSE1456 | Breast cancer | OS | 0.012975 |
| GSE4271 | Brain cancer | OS | 0.013415 |
| GSE3494 | Breast cancer | DSS | 0.01436 |
| GSE31210 | Lung cancer | OS | 0.015052 |
| GSE1456 | Breast cancer | RFS | 0.017259 |
| GSE1456 | Breast cancer | RFS | 0.019606 |
| GSE2658 | Blood cancer | DSS | 0.021756 |
| GSE2658 | Blood cancer | DSS | 0.022514 |
| GSE17537 | Colorectal cancer | DSS | 0.022708 |
| GSE9195 | Breast cancer | DMFS | 0.024236 |
| GSE16131 | Blood cancer | OS | 0.027672 |
| GSE1456 | Breast cancer | DSS | 0.029055 |
| GSE19615 | Breast cancer | DMFS | 0.029679 |
| GSE2658 | Blood cancer | DSS | 0.031383 |
| GSE16581 | Brain cancer | OS | 0.032206 |
| GSE17537 | Colorectal cancer | DFS | 0.033958 |
| GSE31210 | Lung cancer | RFS | 0.034197 |
| GSE2658 | Blood cancer | DSS | 0.035098 |
| GSE1456 | Breast cancer | DSS | 0.037992 |
| GSE19234 | Skin cancer | OS | 0.039527 |
| GSE11595 | Esophagus cancer | OS | 0.039893 |
| GSE19234 | Skin cancer | OS | 0.040044 |
| GSE1456 | Breast cancer | DSS | 0.040637 |
| GSE17537 | Colorectal cancer | OS | 0.042439 |
| GSE2990 | Breast cancer | DMFS | 0.043585 |
| GSE1456 | Breast cancer | DSS | 0.043756 |
| GSE5287 | Bladder cancer | OS | 0.044084 |
| GSE4922 | Breast cancer | DFS | 0.046047 |
| GSE19234 | Skin cancer | OS | 0.046294 |
| GSE31210 | Lung cancer | OS | 0.046761 |
| GSE19234 | Skin cancer | OS | 0.047191 |
| GSE19234 | Skin cancer | OS | 0.04904 |

**Supplementary Table 4. The correlation between the PTBP1 expression and drug sensitivity in diverse cancer cells lines analysis by the CTRP database. (cor<-0.3)**

| symbol | drug | cor | fdr |
| --- | --- | --- | --- |
| PTBP1 | GSK-J4 | -0.40136 | 0.004796 |
| PTBP1 | GSK461364 | -0.38708 | 4.61E-27 |
| PTBP1 | BRD-K30748066 | -0.37105 | 0.021235 |
| PTBP1 | docetaxel | -0.36427 | 3.94E-11 |
| PTBP1 | CD-437 | -0.36398 | 7.23E-24 |
| PTBP1 | teniposide | -0.36133 | 9.43E-13 |
| PTBP1 | COL-3 | -0.35398 | 2.24E-16 |
| PTBP1 | cytarabine hydrochloride | -0.35112 | 1.03E-22 |
| PTBP1 | BI-2536 | -0.34863 | 6.75E-23 |
| PTBP1 | tivantinib | -0.34204 | 1.32E-10 |
| PTBP1 | triazolothiadiazine | -0.33096 | 1.34E-20 |
| PTBP1 | narciclasine | -0.32927 | 2.98E-19 |
| PTBP1 | SB-743921 | -0.32873 | 2.57E-20 |
| PTBP1 | clofarabine | -0.32856 | 4.14E-20 |
| PTBP1 | GW-843682X | -0.3271 | 1.04E-17 |
| PTBP1 | topotecan | -0.32615 | 7.44E-20 |
| PTBP1 | BRD-K70511574 | -0.32244 | 7.85E-19 |
| PTBP1 | bafilomycin A1 | -0.32189 | 0.435044 |
| PTBP1 | vincristine | -0.32103 | 9E-20 |
| PTBP1 | decitabine | -0.32021 | 5.85E-19 |
| PTBP1 | NVP-231 | -0.31863 | 3.88E-18 |
| PTBP1 | barasertib | -0.31705 | 8.19E-18 |
| PTBP1 | necrosulfonamide | -0.31566 | 1.45E-12 |
| PTBP1 | 3-Cl-AHPC | -0.31551 | 7.33E-18 |
| PTBP1 | indisulam | -0.31388 | 6E-17 |
| PTBP1 | PHA-793887 | -0.31364 | 3.92E-18 |
| PTBP1 | MK-1775 | -0.31217 | 3.52E-17 |
| PTBP1 | ceranib-2 | -0.31146 | 4.82E-18 |

Supplementary Table 5. The pathological characteristics of patients with LAUD

| Patients characteristics | No. (%) |
| --- | --- |
| **LUAD patients** |  |
| **Age(years)** |  |
| ≤50 | 9 (56%) |
| ＞50 | 7 (44%) |
| **Gender** |  |
| Male | 6 (60%) |
| Female | 10 (40%) |
|  |  |
|  |  |
